# Advances in nanotechnology applied to natural products

**DOI:** 10.3762/bjnano.17.36

**Published:** 2026-04-24

**Authors:** Douglas Dourado, Fábio Rocha Formiga, Éverton do Nascimento Alencar, Franceline Reynaud

**Affiliations:** 1 Department of Immunology, Aggeu Magalhães Institute (IAM), Oswaldo Cruz Foundation (FIOCRUZ), 50670-420, Recife, PE, Brazil; 2 Faculty of Medical Sciences (FCM), University of Pernambuco (UPE), 50100-130, Recife, PE, Brazil; 3 Laboratory of Micro and Nanostructured Systems (LASMINano), Federal University of Mato Grosso do Sul (UFMS), 79070-900, Campo Grande, MS, Brazil; 4 Université de Lorraine, CITHEFOR EA3452 - Nancy, France

**Keywords:** drug delivery, nanomaterials, nanomedicine, natural compounds, sustainability

Natural products have long played a fundamental role in human history [1]. Besides their in natura or phytotherapeutic use, they have become essential sources of bioactive molecules for various areas (i.e., pharmacological, cosmetic, food, and biotechnological), offering a plethora of structural classes and plant-derived secondary metabolites. These include flavonoids, terpenoids, alkaloids, polyphenols, coumarins, lignans, carotenoids, natural peptides, and fixed and essential oils [2].

These molecules exhibit a wide variety of biological properties, thoroughly described in the literature, including: (i) antioxidant, (ii) anti-inflammatory, (iii) antimicrobial, (iv) vector-control capacity (e.g., larvicidal and repellent properties), and (v) antitumor [2]. However, despite their chemical and functional diversity, they have significant limitations, such as (a) low solubility, (b) thermal or photochemical instability, (c) rapid metabolic degradation, (d) reduced permeability, (e) volatility, and (f) unpleasant organoleptic characteristics. These attributes have compromised the potential of natural products towards their extensive application in diverse fields [1].

In this context, nanotechnology emerges as a promising strategy to overcome limitations of bioactive compounds, and improve their efficacy in various biomedical scenarios. Nanoformulations can protect unstable compounds, improve sensory acceptability, modulate the release of cargos, increase bioavailability, improve tissue penetration, and enhance functional biological performance, often by improving stability and delivery [1]. Thus, the association between nanotechnology and natural products not only drives therapeutic innovation but also redefines the role of these compounds across multiple contemporary technological domains.

In this thematic issue, we bring together advances from original full research, letter, and review articles examining how nanostructured systems can enable the development of safer, more stable, and more effective formulations containing natural products. The collection spans lipid-based, polymeric, hybrid, and inorganic platforms, reflecting the interdisciplinarity of the field, from nanosystem synthesis and physicochemical characterization to biomedical, pharmaceutical, cosmetic, and environmental applications. Among the nanostructures, lipid-based carriers, including nanoemulsions, microemulsions, and liposomes, are prominent across both experimental investigations and comprehensive reviews.

Nanoemulsions are kinetically stable nanoscale systems (typically ≈20–200 nm, sometimes extending into the submicron range) formed by two immiscible liquids and stabilized by surfactants; they can be formulated as oil-in-water (O/W) or water-in-oil (W/O) systems [3]. These carriers have been widely used to deliver natural products, particularly essential or fixed oils and their isolated constituents, for a wide range of applications. In this thematic issue, contributions examine nanoemulsion-based formulations in anti-inflammatory, antiparasitic, and vector-control contexts. By protecting sensitive constituents from physicochemical degradation and promoting delivery to sites of action, nanoemulsions can enhance the functional performance of natural bioactives [4]. Their organization into nanodroplets increases interfacial area and contact with biological surfaces, improves the apparent solubilization of predominantly lipophilic compounds, and can reduce undesirable organoleptic characteristics [5–6].

In inflammatory settings, several preclinical studies have reported lower levels of pro-inflammatory mediators with nanoemulsified oils than with the corresponding unprocessed ones (in natura) [7–8]. In this collection, contributions explore the applications of these formulations in the context of local edema and ocular inflammation. Beyond anti-inflammatory applications, nanoemulsions containing natural products have also been widely investigated for their antiparasitic activity [9]. Notably, some of the studies featured here suggest that nanoemulsion droplets can be internalized by infected macrophages, increasing local exposure at the host–parasite interface and thereby enhancing leishmanicidal activity compared with nonformulated oils. The issue also includes research illustrating the potential of nanoemulsions for vector control. This subject particularly highlights the potential of the high oil interface area of nanoemulsions in enhancing penetration through the cuticle of *Aedes aegypti* larvae [10] and reducing oil volatility and improving stability, enabling their use in repellent formulations [11].

Similar to nanoemulsions, microemulsions have also been widely applied in formulations containing natural products, for both topical and systemic purposes. Microemulsions (10–100 nm) are thermodynamically stable, isotropic systems composed of oil, water, surfactants, often with a co-surfactant, and typically requiring higher surfactant–co-surfactant concentrations than nanoemulsions [12]. As carriers for essential oils, fixed oils, and other lipophilic natural metabolites, microemulsions can improve physicochemical stability, increase apparent bioavailability, and modulate biological responses. In systemic delivery, microemulsions have been reported to enhance the absorption and therapeutic performance of natural compounds, with associated reductions in metabolic alterations and oxidative stress in experimental models [13]. In topical applications, they can promote skin permeation, and controlled release and support formulation robustness, including those intended for transdermal and ocular administration, thereby consolidating microemulsions as versatile and practical platforms for the development of formulations based on natural products [14–15].

Among lipid-based vesicular nanosystems, liposomes are widely investigated for the delivery of natural products. Liposomes consist primarily of phospholipids (often with cholesterol or others sterols) arranged in one or more bilayers, while additional surface modifications (e.g., polymer or polysaccharide coatings) can be used to tailor stability and biological interactions [16]. These vesicles are attractive due to their biocompatibility and capacity to encapsulate both hydrophilic and lipophilic substances [17].

Liposomes have been explored in diverse biomedical applications, including cancer therapy, where they may be designed as conventional liposomes or as immunoliposomes functionalized with antibodies or ligands to promote targeted delivery to tumor cells or components of the immune microenvironment [18]. Combining liposomes with natural products and co-loaded therapeutic agents has enabled the development of multifunctional platforms, which can improve cellular uptake, enhance tumor penetration, reduce systemic side effects, and integrate therapeutic and diagnostic functions [19].

Beyond lipid-based systems, polymeric nanosystems have emerged as versatile nanoplatforms for the delivery of natural products, offering structural stability, tunable release profiles, and controllable surface properties [20]. Polymeric nanoparticles, in particular, can protect bioactive compounds, potentially reduce off-target toxicity, and enhance specific biological responses, broadening the use of natural products in areas such as biological product development, immunization, and vector control [21–22]. In addition, polymeric micellar systems, such as poloxamer-based systems, have been explored to improve the solubility and dispersion of poorly water-soluble natural compounds, underscoring the importance of physicochemical approaches in the development of nanostructured formulations [23–24].

More recently, hybrid nanosystems have emerged as promising strategies for natural product delivery and application. Structures that combine polymer–lipid interfaces, such as core–shell nanofibers, have been proposed for the simultaneous transport of hydrophilic and lipophilic bioactives, leveraging compartmentalization to combine material advantages and enable multifunctional controlled-release strategies [25].

Beyond organic nanocarriers, research has increasingly extended to inorganic nanomaterials, particularly metallic nanoparticles. Metallic nanoparticles, typically defined as structures with diameters between 1 and 100 nm, exhibit distinctive electrical, optical, and magnetic properties that support a broad range of applications [26]. They can be obtained via green synthesis using natural products, such as plant extracts or other biological agents rich in reducing and stabilizing metabolites (e.g., polyphenols and flavonoids), supporting more sustainable routes that avoid harsh or toxic reagents and can enhance colloidal stability and modulate biological performance [27]. Moreover, metallic nanoparticles have shown promise in antiseptic and therapeutic contexts, including the prevention of biofilm formation and infection in dentistry, integration into restorative or regenerative dental materials, and modulation of biological responses in clinical treatments [28].

Overall, the contributions in this thematic issue highlight the significant potential of nanotechnology to overcome limitations of natural products, thereby enabling a wide range of effective applications, as noted in this editorial. By covering a broad range of nanostructured systems, this collection reflects the diversity of strategies currently used to improve the stability, bioavailability, targeting, and functional biological performance of natural products. The contributions presented highlight the inherently interdisciplinary character of the field, bridging fundamental physicochemical approaches with biomedical, pharmaceutical, cosmetic, dental, environmental, and other technological applications. We hope this thematic issue will stimulate further research, foster interdisciplinary collaboration, and inspire innovative nanotechnology-based solutions that can expand and consolidate the use of natural products across scientific and applied contexts.

Douglas Dourado, Fábio Rocha Formiga, Éverton do Nascimento Alencar and Franceline Reynaud.

Recife, Campo Grande, and Lorraine, March 2026

## Data Availability

Data sharing is not applicable as no new data was generated or analyzed in this study.
